# Traumatic fractures in an early 19th century museum skeleton suggest the homicide of an old Munich character: the history of “Finessensepperl” (Finesse Joseph)

**DOI:** 10.1007/s12024-024-00811-w

**Published:** 2024-04-12

**Authors:** Andreas G. Nerlich, Stephanie Panzer, Christine Lehn, Jan Friederichs, Oliver K. Peschel

**Affiliations:** 1 Department of Forensic Histopathology, Paleopathology and Mummy Research, Institute of Legal Medicine, Ludwig-Maximilians Universität, Nussbaumstr. 26, D-80336 München, Germany; 2 Department of Radiology, Berufsgenossenschaftliche Unfallklinik Murnau, Murnau, Germany; 3 Institute of Biomechanics, Berufsgenossenschaftliche Unfallklinik Murnau and Paracelsus Medizinische Universität, Salzburg, Austria; 4 Department of Trauma Surgery, Berufsgenossenschaftliche Unfallklinik Murnau, Murnau, Germany

**Keywords:** Skeleton, Trauma, CT scan, Stable isotope, Histology

## Abstract

The well preserved skeleton of Joseph Huber, a very well-known historical character of the 19th century Munich, also nicknamed “Finessen-Sepperl”, is the starting point of the reconstruction of life and death of this historical individual. He was known as a *postilion d´amour* (love’s messenger) of the Royal Bavarian capital with numerous comments and anecdotes and a few biographical sketches that indicate he remained well until the last few years of his life where requests for his duties lessened. The skeleton shows a small-sized male individual with almost complete loss of teeth, but otherwise very well-mineralized bone, having suffered from three episodes of trauma – an old-healed incomplete femoral neck fracture leading to severe osteoarthrosis, a clavicle fracture of the medial third with a few weeks old callus formation, and fresh serial rib fractures along with severe skull trauma with fractures of the os temporale and petrosum, presumably leading to intracranial bleeding and finally death. The type and distribution of these latter two injuries are in agreement with a murderous attack – which was retrospectively reported several years after his death, while the old-healed femoral neck fracture may have caused reduction in Joseph´s walking activities but not reduced requests for his services. Paleopathology not only identifies the terminal decline, but also previous diseases of this Old Bavarian character and thereby completes his story.

## Introduction

In the early 19th century, the preparation and conservation of complete human skeletons was a frequent practise in order to obtain and preserve teaching material for medical education and scientific analysis. This holds also true in Munich where the national Bavarian university (Ludwig-Maximilians-University) had started to form an appropriate collection following the transfer of this university to the royal Bavarian capital in the year 1826 [1].

Beyond the collection of clearly pathological specimens locally well-known characters also seem to have been a target for this collection. Therefore, the death of an historical Münchner, the so-called Finessen-Sepperl (literally “Finesse Joseph” in a sense of “Discreet Joe”), lead to the preparation of his skeleton and its inclusion into the anatomic collection.

The skeleton of the Finesse Joseph has luckily survived to this day. This enabled a scientific investigation in order to obtain more data on his life, disease and death. This is of interest as there exists oral and written reports of this early 19th century Munich character with, however, only limited information on his biography [2].

During this evaluation, we obtained data on several repeated traumatic events at various time points of his life – including some osseous injuries that are almost certainly associated with his death. Beyond this, some data on his overall physical condition could be obtained. The type and localization of the signs of trauma will be described here along with the most obvious interpretation of their cause. Accordingly, we provide evidence that the “Finessen-Sepperl” most likely was the victim of a murderous attack which finally led to his death.

## Life and history of the Finessen-Sepperl

**Fig. 1:**
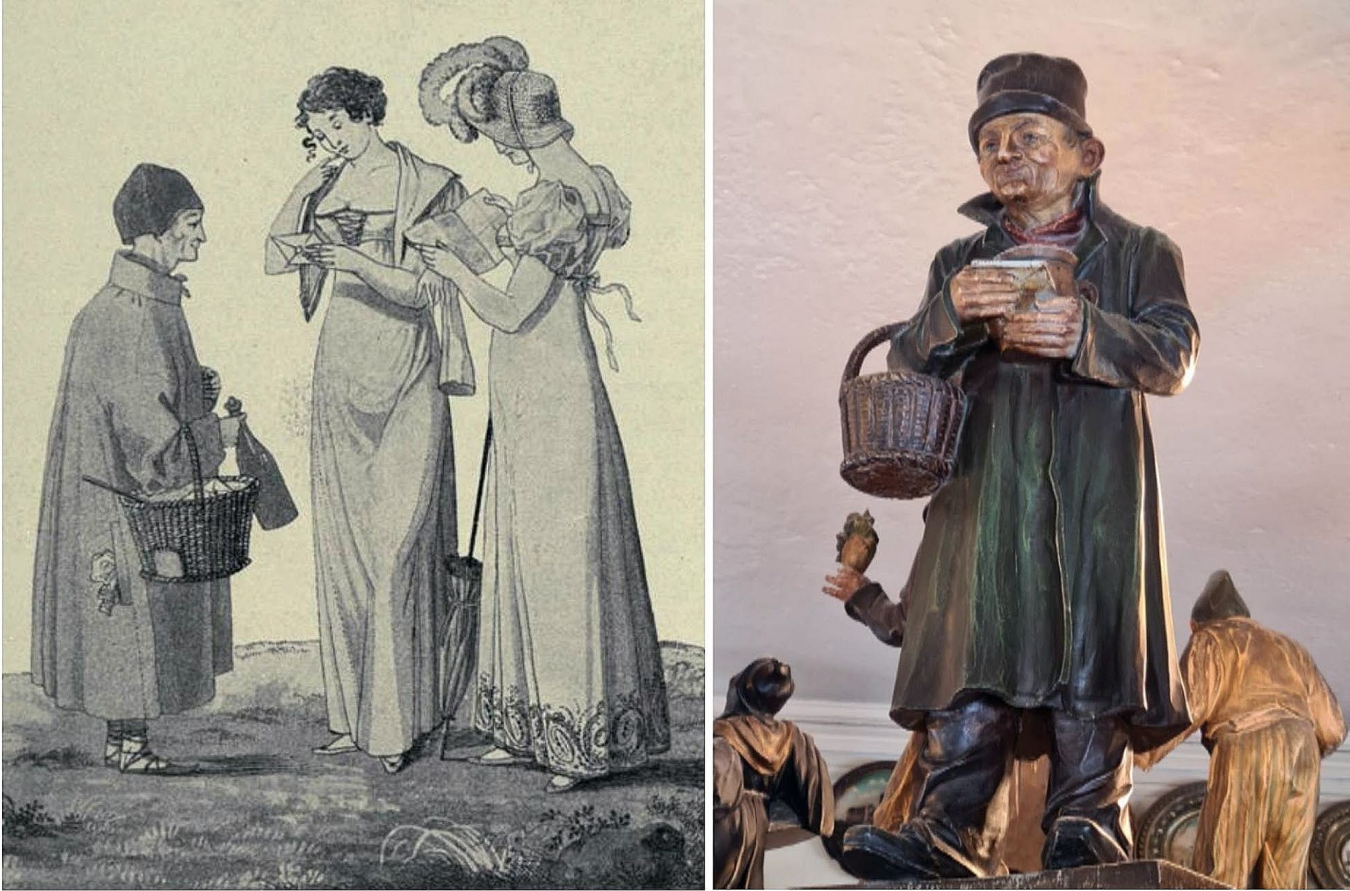
The “Finessen-Sepperl” with his typical appearance as a postilion d´amour. right: lithography by Voelz, representing a scene of the 1810s (printed in 1904); right: small figurine, previously part of the Munich town hall

Finessen-Sepperl was born under the name of Joseph Huber in Munich into a working class family; no information exists either about his exact place or date of his birth. The records of a later official state registry (founded in 1824) suggest that he was born in 1776 [3], most likely in down-town Munich. Nothing is recorded about his life during his youth and early adulthood. There are no data on his education. The first public notes that mention him are in local newspapers. In 1810 he was described as a tiny man with a typical outer appearance wearing mostly a cone-like cap, a caftan and a brass-cross around his neck. Joseph´s main job was the secret transport of messages (as a *postilion d´amour*), exchanging those messages between lovers within the city, for which he received small fees (Fig. 1). This business was facilitated through Joseph´s daily routine of walking through all the streets of Munich, and having open access to all houses, including those of upper class citizens and aristocrats and even the royal residence. Since Joseph always carried a punnet with a double-layered bottom with him, he was able to transmit the messages secretly in this hiding place [2].

Besides this, Joseph was accepted as a permanent member of the city community being involved in all kinds of festivities (e.g. carnival etc.) leading to numerous comments and anecdotes about tricks and jokes. During his final years, it was reported that the upcoming official postal services inhibited Joseph from performing his business adequately. In later descriptions of his life, it was even suspected that he fell into poverty. Furthermore, these later reports describe an assault by an unknown aggressor; the looter tried to steal the little fortune that he had collected.

On March 26th 1829 Joseph was admitted to the Munich General Hospital since “he had fallen onto his shoulder” [4]. Despite quickly recovering, approximately 4 weeks later he had to be readmitted to the Hospital when he sustained a second fall for an unknown reason [5]. This time he did not recover and died on the 26th April 1829. The official death certificate states “senility” as the cause of death [6]. While the soft tissues of his body were soon buried, his skeleton was prepared at the Anatomical Institute where this was kept before being transferred to the Anatomo-Pathological Collection and preserved to this day. Currently the skeleton is part of the Siegfried-Oberndorfer Collection for Pathology, Munich Clinic Schwabing (former director: Prof. Dr. A. Nerlich; without specific registration number).

## Materials and methods

The skeleton was prepared immediately after death including the preservation of the tight connective tissues of the joint capsules so that only some minor artificial fixings, such as the skull and the cervical region of the vertebral column, along with a metal frame for his standing position, were necessary. Although there exists no written information, we assume that the skeleton was prepared by manual defleshing followed by maceration with skin beetles (*Dermestes lardarius*) which leaves not only bone, but also cartilage and the tight connective tissue of the joint capsule intact. This technique was usually used in historic periods [7].

The skeleton was first inspected macroscopically, then subjected to a complete CT- scan (Siemens Somatom, 120 V, slice thickness 0.6 cm). The body height of the skeleton was initially directly measured. Since, however, the preparation of the skeleton obviously has led to some shrinkage (particularly of the intervertebral discs) and the soft tissue surface was lacking, the body length *intra vitam* was estimated using measurements of the long bones (femora, tibiae, humeri) as usually performed in bioanthropology [8]. The obtained individual values were averaged. Finally, some small tissue samples were obtained for stable isotope analysis and selected tissue histomorphology. For stable isotope analysis, the tissue samples were taken from the only available tooth root, along with bone from compact bone of the central right femur, one rib and a small sample from the skull. This sampling was selected since it provides a broad range of time periods: Depending on different formation times and remodelling rates, the body tissues examined represent different stages of life: childhood/ adolescence (dentin), youth until death (skull), the last 30 years of life (femur) and the last 10 years of life (rib). For the joint capsule, information from the last few years of life can be deduced [9]. The extraction of collagen and stable isotope analyses of carbon, nitrogen, sulphur and hydrogen followed the usual protocols [10]. For the preparation of the histological analyses, the bone samples were rehydrated, fixed, decalcified and embedded into paraffin wax as previously described [11].

## Results

**Fig. 2:**
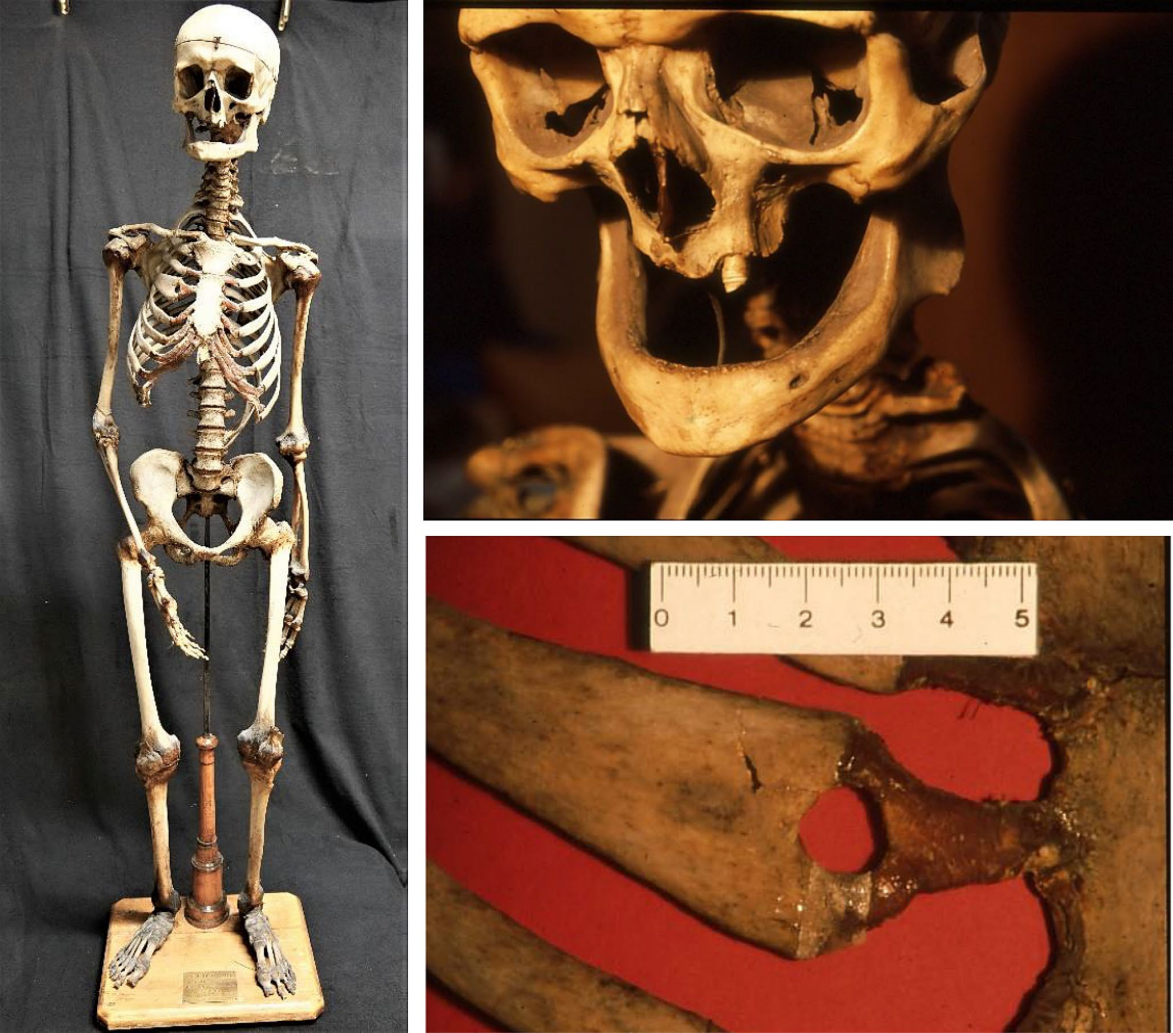
The skeleton of the “Finessen-Sepperl”: The complete skeleton (left panel), the massive loss of dentition (upper right panel) and the right 3rd bifid rib (lower right panel)

**Fig. 3:**
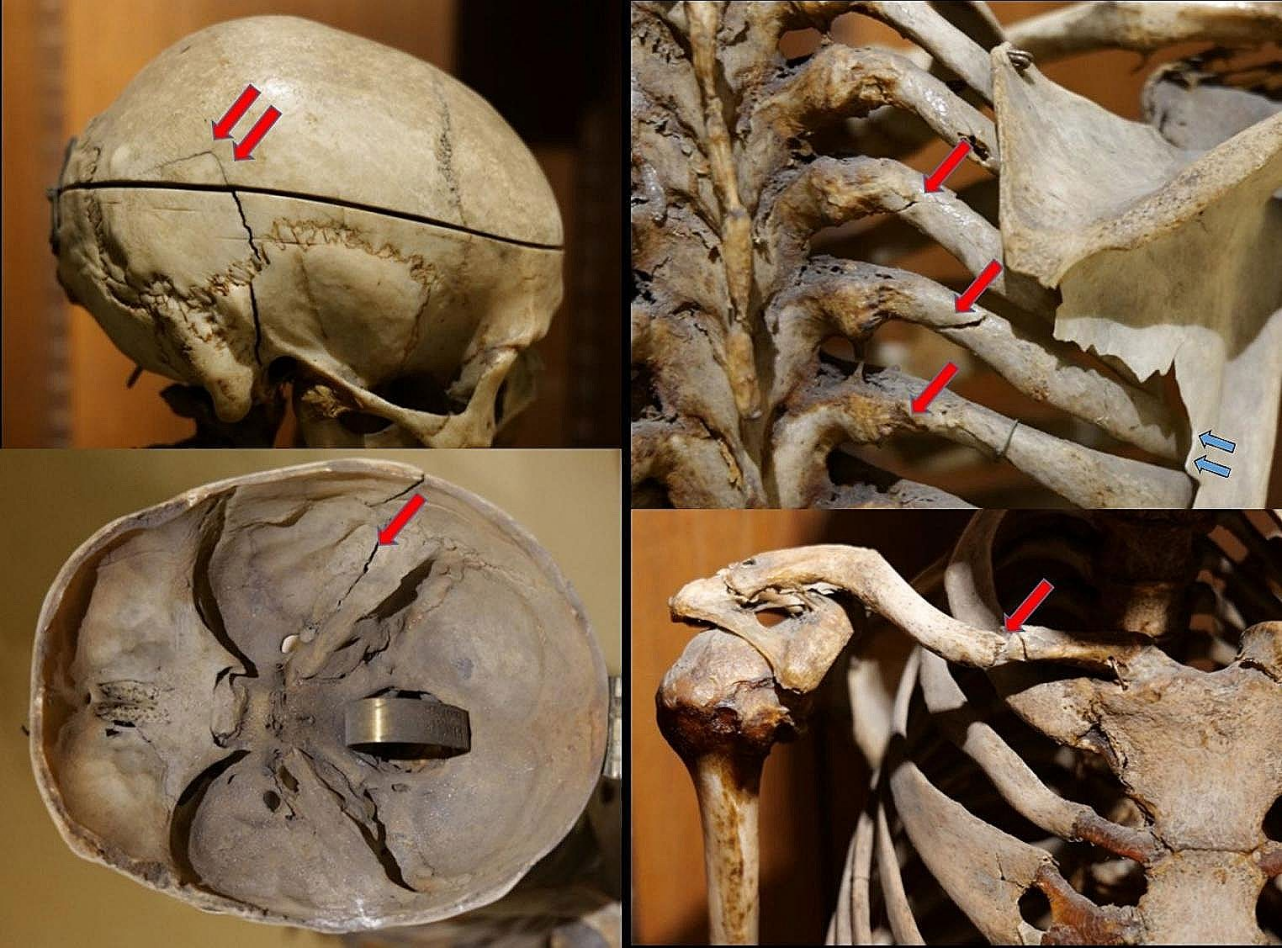
The trauma sequelae: The skull trauma (left panels), serial rib fractures (upper right panel), the midshaft clavicular fracture (lower right panel). Red arrows show the fractures; the blue arrows in the upper right panel show the whitish discoloration of the scapular bone suggesting bone breaks of this bone occurring significantly after the preparation of the skeleton

Macroscopically, the mounted skeleton is that of an advanced-aged male individual; the skeleton has a height of 145 cm. Long bone determinations indicate a living body height of 155 cm +/- 2 cm (mean +/- 1SD). The bones show a proportionate reduction in size, but are otherwise of regular form (Fig. 2) and seem very well mineralized. There exist two epigenetic variations: a persistent metopic suture and a doubling of the distal (sternal) end of the 3rd right rib (bifid rib) (Fig. 2). Pathological features affect the teeth and three body regions show obvious sequelae of trauma. With respect to the teeth, there is an extensive intravital loss with closure of all the dental alveoli except one tooth, region 23 (upper left canine) which is significantly eroded but without other pathology (Fig. 2). Accordingly, *pre mortem* loss of the teeth (with the exception of tooth 23) over a prolonged period of time can be assumed. As to the trauma sequelae there is an obvious fracture line at the skull (right temporal bone) extending into the skull base, a series of rib fractures of the right ribs 3–6 medio-dorsally and a fracture of the medial third of the right clavicle (Fig. 3). These suggest a post-traumatic origin that deserved further investigation.

**Fig. 4:**
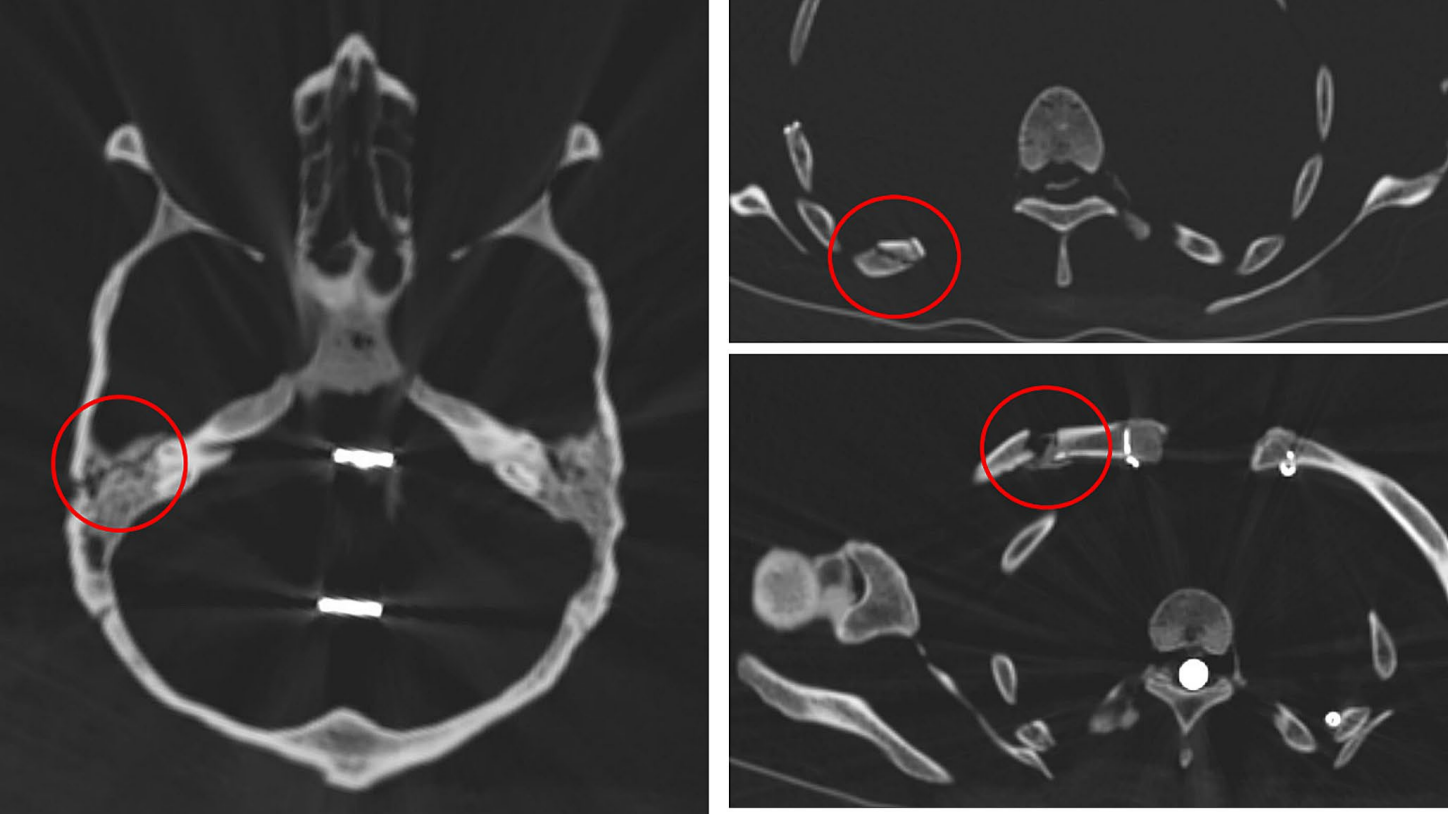
CT-scans of the skull trauma with lesion of the os petrosum (left), rib fracture (upper right) and the clavicle fracture (lower right)

**Fig. 5:**
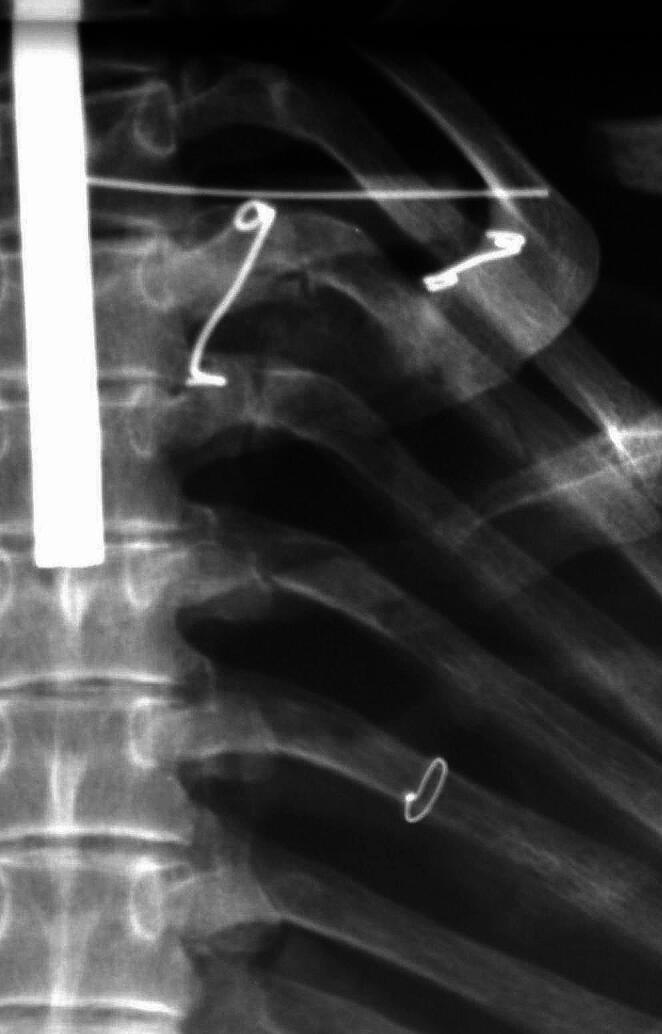
Chest x-ray of the right upper thorax. This shows the ring-like wire fixation of rib 6 identical to other post-mortem fixations of the skeleton (rib, apical scapula, vertebra)

**Fig. 6:**
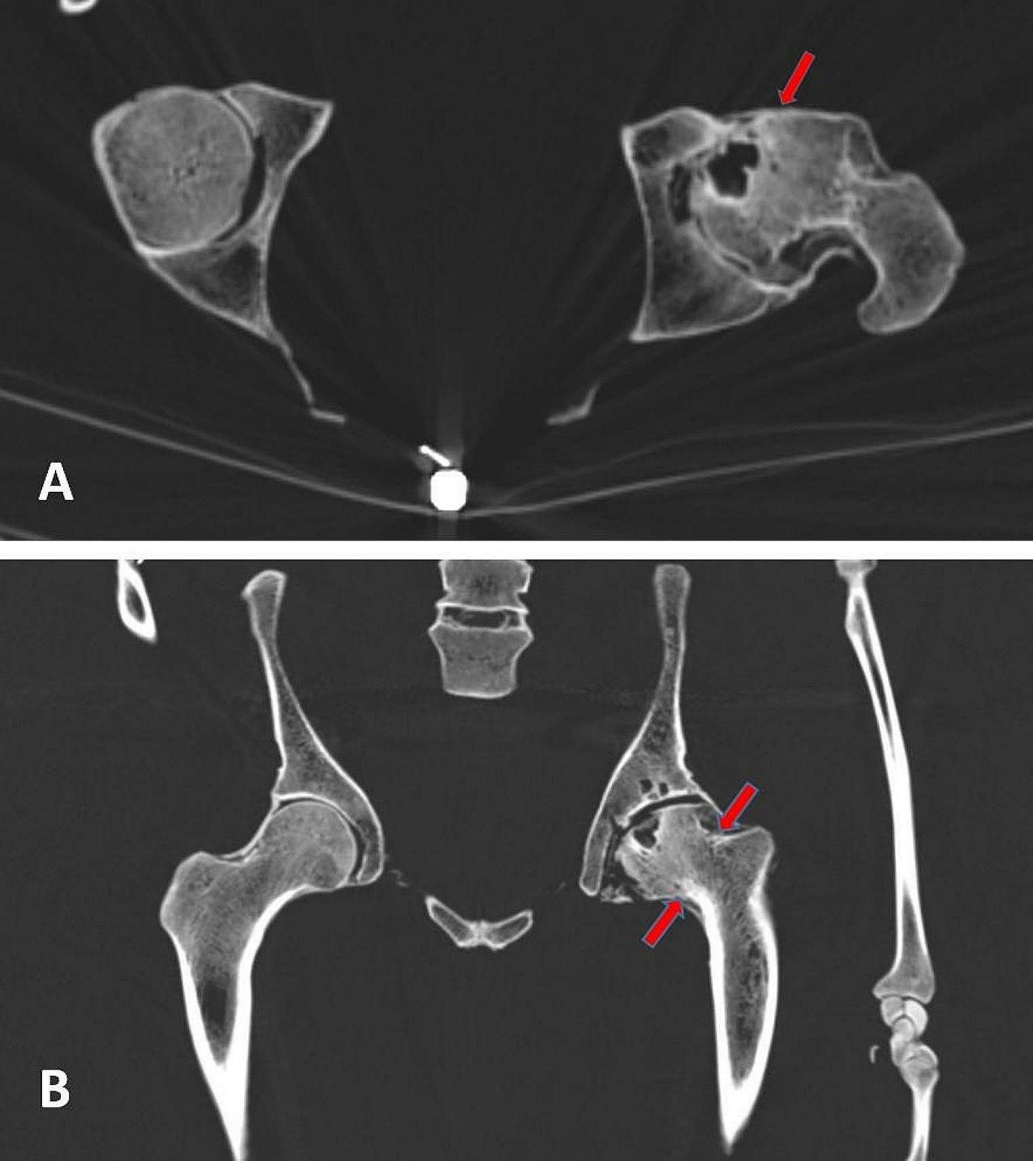
CT-scan of the left hip joint with severe posttraumatic arthrosis. (**A**) Horizontal section through both hip joints shows severe osteoarthrosis of the left hip joint. (**B**) On a coronal section the impaction of the femoral head strongly suggests an old-healed fracture (between the red arrows). This coronal section also demonstrates the coxa vara position of the left femoral head

This further investigation comprised first of a whole body CT-scan as indicated before. The scans confirmed the excellent state of mineralization of all bones, the almost complete loss of dentition (without further jaw pathology, including a lack of any trauma residue of the mandible or maxilla) and the trauma sequelae described above. In addition, the skull trauma showed radiologically a much more extended length running, not only in a square-like shape at the right parieto-temporal bone, but running deeply into the temporal bone down to the carotid canal with, however, lack of displacement (Fig. 4); accordingly, a direct laceration of the carotid artery is unlikely. Despite this, significant intracranial (epi-/subdural) bleeding is possible since intracranial blood vessels may have been affected by the trauma. There is no bone reaction at this fracture site. The serial rib fractures are also without bone reaction (Fig. 4). The localized fractures of only four ribs argues in favour of a specific trauma affecting only this skeletal region. Most of these rib fractures are in a stable position. The fracture of rib 6, however, obviously was unstable at the time of preparation of the skeleton so that it had to be fixed by a small round metal wire (Fig. 5). This fixation was performed in an identical manner as other small fixations of the skeleton (e.g. the scapula) so that it is plausible that this was done during the preparation of the skeleton. This excludes a later post-mortem break, e.g. by mishandling of the prepared skeleton. Nearby these rib fractures, the right scapula shows some defects. Since these, however, show focal whitish surface discoloration, we assume that these may have occurred much later than the preparation of the skeleton (see Fig. 3). In contrast, the clavicle fracture shows a bone reaction with callus formation and incipient bone growth so that this fracture has an older age than skull and rib traumata (Fig. 4). Finally, the CT scans reveal a severe arthrosis of the left hip joint with deformation of the femoral head, narrowing of the joint space, reactive osteosclerosis and multiple subchondral cysts (including sclerosis and cyst formation of the acetabulum) (Fig. 6). The CCD-angle (centrum-collum-diaphysis angle) ranges at 108° indicating a coxa vara (as compared to normal 126° on the right side). This severe joint arthrosis seems most likely to be the result of an old fracture of the femoral neck without severe displacement, but with impaction of the head into the shaft. This must have occurred long before death and may have led to major disabling of the left hip joint. The trauma mechanism must have been a severe blunt trauma long before his death of unknown cause.

**Fig. 7:**
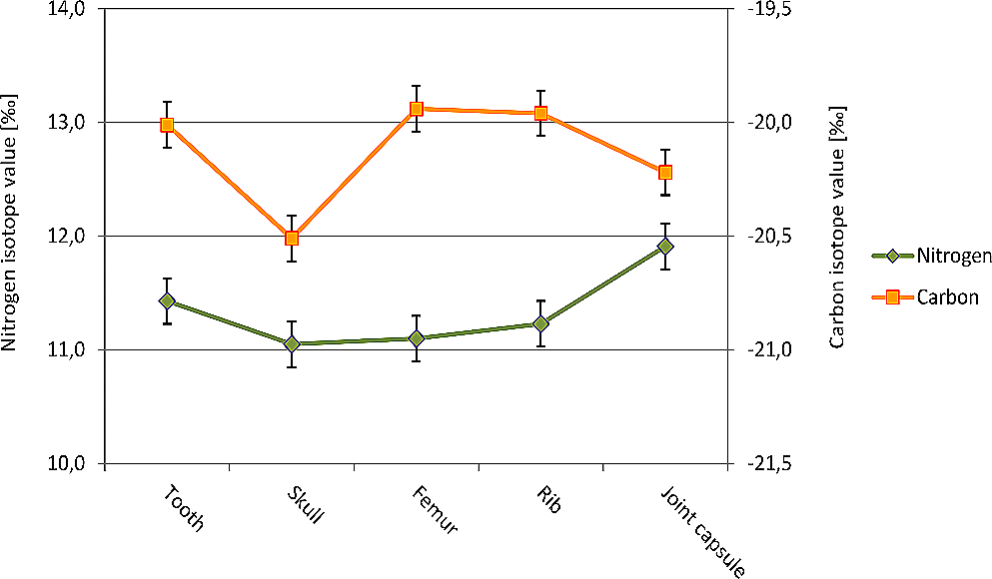
Stable isotopic values of nitrogen and carbon in the collagen samples of various body tissues of the Finessen-Sepperl

The stable isotope analyses of the collagen from different body tissues samples revealed similar nitrogen as well as carbon isotope values of the tooth and bone samples (11.1 to 11.4‰, resp. -20.5 to -20.0‰), and 11.9‰ or -20.2‰ for the joint capsule (Fig. 7). The sulphur isotope values were in the range from 5.7 to 7.3‰, and the hydrogen isotope values resulted between − 43 and − 19‰.

Since the formation times and remodelling rates of various skeletal elements and the joint capsule connective tissue are different, the body tissues examined represent different stages of life: childhood/ adolescence are represented by dentin, youth until death by the skull bone sample, the last 30 years of life are covered by the femur bone sample and the last 10 years of life by the more cancellous bone of the rib. For the joint capsule, information from the last few years of life can be deduced [9]. In general, the isotope pattern shows only slight differences between childhood/adolescence and last stage of life.

Finally, the histomorphology of bone tissue shows a well formed cell-free osseous matrix without evidence for loss of bone, enhanced bone-turn over or any relevant metabolic disturbance.

## Discussion

The very well-preserved skeleton of the famous Munich historic character Joseph Huber, vulgo “Finessen-Sepperl”, is a rich source for a historic, but also medical (re-)examination of this early 19th century individual. Despite his widespread perception in public life of 19th century Munich, there exist only very few official records on his life, such as the official registry entry and the death certificate – and a few contemporaneous notes in several daily newspapers. Almost all other reports dealing with him are comments and stories with limited value for historical research, most of which have been written years or even decades after his death.

**Fig. 8:**
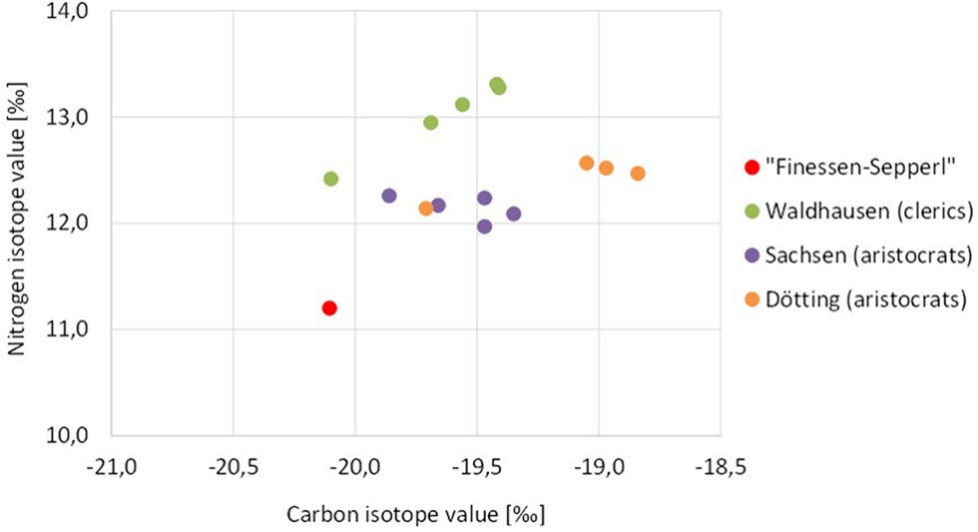
Carbon and nitrogen isotope values of the Finessen-Sepperl compared to clerics (Stift Waldhausen, Upper Austria, AD 1640–1690 *n* = 4) and aristocrats from the 19/20th century (“Sachsen”: family of baronets von Könneritz, Erdmannsdorf, AD 1820–1930, *n* = 5; “Dötting”: family of baronets von Jordan, Dötting/Bavaria, AD 1815–1859, *n* = 4) indicate his relatively low nutritional status

Valuable information is obtained from stable isotope analyses in various tissues (dentin, bones, joint capsule) that may provide an over-all information on the quality of his diet and, indirectly, his social status and personal resources during various periods of life [12]. From traditional written sources we know that Joseph mostly lived from daily donations from numerous middle-class and aristocratic households plus a little income as a *postilion d´amour* [2]. Since the relative values for the stable isotopes analysed, especially that for nitrogen isotope, did not differ significantly between dentin (youth), skull bone, femur and rib bone collagen (adulthood until the last years of life) there is no evidence for major changes in the composition (and thereby the “quality”) of his food during his lifetime. When compared to other individuals/ social groups, the composition of his diet with respect to the amount of animal protein in his diet, was lower than for contemporary aristocrats or clergymen (friars) (see Fig. 8). In this respect, it is noteworthy that Joseph had lost almost all his teeth a long time before death. The reason therefore remains unclear. A posttraumatic loss is possible but not proven. X-rays and CT-scans do not show trauma residues so that extensive tooth loss following periodontitis is possible. However, the only remaining tooth does not show any evidence therefore. In general, however, there is no evidence for severe disease impact or starvation periods; accordingly, long-term vitamin deficiencies (scurvy, osteomalacia) as well as chronic anaemia can be ruled out. This is confirmed by the histopathological analysis of bone samples. Finally, the somewhat increased nitrogen isotope value of the joint capsule may be associated with a change in Sepperl´s food composition, but does not necessarily indicate an emaciation of Sepperl´s body during the last stages of his life [13], since some differences in the collagen amino acid compositions from tooth/bone and the joint capsule have to be taken into account.

Beyond this information the skeleton shows several trauma sequelae that seem to have been relevant to Joseph´s life. Their morphologies suggest three time periods of genesis [14]: the presumed left side femoral neck fracture occurred long time before death, since the resulting coxa vara deformation developed into severe hip joint arthrosis. Accordingly, the underlying trauma must have happened at least several years before Joseph´s death and may correlate with a reduction in his daily prolonged walking through Munich´s streets and also, possibly, with a slightly poorer nutritional supply as evidenced by the stable isotope analysis. The second episode of trauma must have occurred a few weeks before Joseph´s death since the clavicular fracture shows major remodelling including bony callus formation. This trauma would perfectly match with the reported first hospitalization event about 4 weeks before his death when newspaper reports describe a “fall to the right shoulder”. The third event covers the two fracture regions without any bony reaction which obviously occurred around the time of Joseph´s death. Both the multiple fractures of the upper dorsal ribs and the skull fracture affect the right side. There were no other “fresh” trauma sequelae at the skeleton.

To this regard, it is important to discuss whether those breaks happened in the perimortem period (i.e. in a “wet bone status”) or if they were the result of much later post-mortem damage (i.e. “dry bone breaks”). This problem has been widely discussed in both the forensic and bioarchaeological context [18]. A discrimination between those two statuses may indeed be very difficult. Helping criteria may include different staining of fracture ends, desiccation of the bone fragments, loss of bone flexibility and elasticity and finally the type of fracture [15, 16]. In the present setting, the preservation situation in the anatomo-pathological collection clearly rule out frequent problems with many later post-mortem modifications, such as by weathering, geological forces, scavenger modification etc. The fracture zone of all four ribs does not show neither dislocation nor discoloration. The preservation of the fracture zones in their regular anatomical axis suggest bone flexibility and elasticity during the process of fracturing. However, one fracture must have been fixed by a small metal wire which is identical to other metal fixations applied during the process of the skeleton´s preparation. This further supports the presence of those fractures already during the preparation of the skeleton for the collection and thereby a perimortem, but no postmortem damage. Furthermore, with respect to the rib fractures, we can rule out a longer post-mortem break by a fall of the skeleton onto its back which should have also affected the more prominent vertebral processes which remain intact. Finally, only four ribs are affected, but neither the more cranial nor caudal ones so that only a very localized impact can be expected. Taking all these observations together, the rib serial fractures are much more likely perimortem (“wet bone status”) fractures, but not later post-mortem breaks (“dry bone breaks”) [15–18].

There is a different situation with the adjacent scapula which also shows some breaks at its medial margin which, however, show focal whitish discoloration. These latter changes strongly suggest that the scapular defect occurred – at least in some major part – at a much later time, i.e. that there may have been post-mortem breaks which, however, remain unreported in all later descriptions of the skeleton.

The trauma to the right skull came from the side. The square-shaped infraction of the parietal/temporal bone suggest a similarly formed object that may have struck the head. The CT scans further indicate an extension of the fracture to the right petrous bone and the adjacent skull base. Since there is no evidence for a direct disruption of the carotid canal, death from an acute bleed can be ruled out. However, the type and extent of the skull base fracture may have caused intracranial epi- or subdural bleeding with delayed death. This scenario also fits quite well with the clinical course –indicated by newspaper records that Joseph had “an accident” (which was not further described or commented in those records) which led to his hospitalization and ultimately his death within a short period of time (likely several hours).

Assuming the aforementioned scenarios, Joseph was subjected to several trauma events over a long period of time, however, with a final trauma to the right body side. Although we cannot fully exclude a fall to the right side with double impact by accident, the pattern of a trauma from two directions is also well in agreements with a double hit by an attacker. Alternatively, Joseph may have suffered from a blow to the skull and then falling on a shaped object with his thoracic back. Anyway, this would well agree with a later report of a direct attack. Accordingly, it is most likely that the “Finessen-Sepperl” was attacked, possibly during an attempted robbery of the Joseph´s small fortune that he had collected during life time as the *postilion d´amour* of Munich in the early 19th century, and thereby killed within several hours by a severe intracranial bleeding. The excellent preservation of this skeleton, from about 200 years ago, enables us to reconstruct this historical cold case.

## Data Availability

All data are included in the manuscript.
